# Combatting malaria together: key findings from a rapid formative assessment on Royal Thai Army malaria practices and military-civilian collaboration in Sisaket Province, Thailand

**DOI:** 10.1186/s12936-026-05884-2

**Published:** 2026-04-11

**Authors:** Emily Dantzer, Sutchana Tabprasit, Kanokwan Suwannarong, Krisada Jongsakul, Khunakorn Kana, Pratin Dharmarak, Mitra Feldman, Prayuth Sudathip, Jintana Chaiwan, Watcharee Yokani, Min Kramyoo, Kannika Thammasutti, Chris Cotter

**Affiliations:** 1 Malaria Elimination Initiative, Institute for Global Health Sciences, University of California San Francisco (UCSF), 550 16th Street, San Francisco, CA 94143 USA; 2 Research Division, Armed Forces Research Institute of Medical Sciences (AFRIMS), Royal Thai Army, Bangkok, Thailand; 3 SUPA71 Co., Ltd., Bangkok, Thailand; 4 Department of Bacterial and Parasitic Diseases, Walter Reed Army Institute of Research - Armed Forces Research Institute of Medical Sciences (WRAIR-AFRIMS), Bangkok, Thailand; 5 Freelance Consultant, Bangkok, Thailand; 6 Independent Global Health Consultant, Silver Spring, MD USA; 7 Division of Vector Borne Diseases, Department of Disease Control, Ministry of Public Health, Nonthaburi, Thailand; 8 Department of Women’s and Children’s Health, Uppsala University, Uppsala, Sweden

**Keywords:** Malaria, Military, Malaria elimination, Military-civilian collaboration, Royal Thai Army, Thailand, Greater Mekong Subregion

## Abstract

**Background:**

In the Greater Mekong Subregion, militaries constitute a critical but often underserved malaria transmission reservoir, given their high mobility, deployment to endemic areas, and frequent exposure to vectors. In Thailand, the Royal Thai Army (RTA) is a key risk population, yet their malaria practices, perceptions, and the scale and scope of coordination with the Ministry of Public Health (MoPH) are not well understood. A joint military-civilian research team conducted a rapid formative assessment in Sisaket Province, a persistent transmission hotspot, to characterize the unique RTA risk profile and identify opportunities to strengthen RTA-MoPH coordination on malaria elimination efforts.

**Methods:**

Using a mixed-methods design, the research team conducted a five-year (2016–2020) retrospective analysis of Sisaket’s malaria case and program response data, alongside 16 focus group discussions (FGDs) and 17 key informant interviews (KIIs) with RTA and MoPH respondents across all military ranks and health system levels in the province’s three highest-burden districts (Kantharalak, Khun Han, Phu Sing). Qualitative data were collected between December 2021-January 2022 and thematically analyzed using an inductive content analysis approach. Quantitative data were descriptively analyzed using statistical methods to characterize the RTA malaria risk profile and identify and compare recent trends in program response between military and non-military populations in Sisaket Province.

**Results:**

432 military malaria cases were reported in Sisaket between 2016 and 2020, accounting for 18% of the province’s total 2425 cases (with the proportion ranging from 14 to 30% annually). All military malaria cases were male, with a median age of 28 years. 96% of military cases were diagnosed and treated at MoPH facilities, with 41% of cases classified as indigenous and 40% as imported from abroad. Qualitative data were collected from a total of 116 respondents (72 RTA, 44 MoPH) through 16 FGDs and 17 KIIs. Malaria prevention and treatment practices and perceptions among soldiers were largely consistent across the three study districts. RTA-MoPH coordination occurred at all levels through both formal and informal channels and was reported to be improving and expanding by some respondents. Though several areas could benefit from increased collaboration: patient follow-up, border control efforts, vector control, and further capacitation of military medics. The RTA and MoPH both expressed strong interest in strengthening military-civilian coordination; though to better enable this, several identified challenges may need to be addressed, including restricted MoPH access to military sites, lack of continuity in relationships due to frequent RTA rotations, communication constraints, and broader differences in military versus civilian operating procedures.

**Conclusion:**

Strengthening RTA-MoPH coordination towards successfully interrupting malaria transmission in Thailand may require formalizing and standardizing some joint operating procedures, increasing communication and military-civilian touchpoints, and further capacitating the RTA to carry out malaria prevention, diagnosis and treatment, and patient follow-up activities. For Thailand to achieve its goal of nationwide malaria elimination, it is crucial that the RTA as a high-risk population be fully engaged in the country’s malaria control and response efforts.

## Background

Thailand has made significant gains in the fight against malaria over the past decade, with a 91% reduction in cases from 35,910 in 2012 to 3268 in 2021 [1]. Transmission had been interrupted across much of the country, with local transmission occurring in less than 1% of villages, when in 2022 cases surged to over 10,000, driven by increased migration into western Thailand from Myanmar amid the ongoing civil war there [1, 2]. Thailand’s National Malaria Elimination Strategy (NMES) 2017–2026 still calls for malaria elimination nationwide by 2026, with the 1-3-7 case-based surveillance and response strategy identified as a core intervention [3]. Adopted from China, 1-3-7 refers to reporting a malaria case within one day (24 h), investigating and classifying the case within three days, and implementing village-level response measures within seven days towards preventing or controlling an outbreak [3].

Malaria transmission is concentrated in remote, forested areas along international borders with Cambodia, Myanmar, and Malaysia, and in hard-to-reach populations including mobile and migrant populations, forest-goers, plantation workers, ethnic minorities, and border security and military personnel. Owing to their high mobility, frequent exposure to vectors, deployment to endemic areas, and potential for asymptomatic parasite carriage, military populations are at increased risk for malaria, yet are often overlooked by government health services [4]. While militaries have been recognized as a key risk population across the Greater Mekong Subregion, there remains a critical dearth of publicly available data and research on this population [1, 5–7].

Under the Ministry of Defense in Thailand, the Royal Thai Armed Forces is comprised of three branches (Army, Navy, and Air Force) with the Army being most affected by malaria owing to its’ soldiers’ patrol and reconnaissance activities along the country’s forested land borders [8]. Established in 1900, the Army Medical Department (AMED) of the Royal Thai Army (RTA) is the key agency responsible for the policy and provision of military health services [8]. The Ministry of Defense serves as one of four vice-chairs on Thailand’s National Malaria Elimination Steering Committee and is identified in the NMES as among the organizations responsible for scaling up elimination efforts including increasing the capacity and coverage of malaria diagnosis and treatment services [3].

The AMED has aligned malaria prevention and treatment guidelines and related trainings with those of the MoPH, but as of 2023 no specific elimination guidance had been issued by or for the military, which is thought to have led to heterogeneous malaria service delivery across the different RTA forces, divisions, and units [5, 8]. The quality and reach of key malaria interventions are thought to be variable depending on RTA mobility and access to resources including personnel, funding, and supplies at local levels. The AMED encourages RTA divisions and units to work collaboratively with local MoPH facilities and health workers, including educating soldiers on malaria prevention, training military medical staff on diagnosis and treatment, and implementing joint outbreak response measures, although the scale and scope of activities are thought to vary depending on the area, local resource availability, and the policies and functions of the RTA units deployed.

Serving as the catalyst for the present study, previous research on a joint MoPH-RTA malaria outbreak investigation and response in 2017 in Sisaket Province identified the need for stronger military-civilian collaboration; prior to the outbreak, coordination between the MoPH and RTA was reported to be limited [5]. Bordering northern Cambodia, Sisaket Province has a strong military presence and has historically been a transmission hotspot with malaria affecting civilian and RTA populations alike. The RTA was highly affected by the 2017 outbreak, particularly young soldiers with no prior exposure to malaria, with increased RTA mobility along the forested Thai-Cambodian border identified as a key driver of the epidemic [5]. More recently, the number of reported malaria cases in Sisaket has declined significantly, with only 66 cases reported in 2020, as compared to 386 in 2019 and over 800 cases each year from 2016–2018, yet the factors underlying this reduction in transmission are not well documented [1]. Having achieved and thus far sustained this dramatic decline in cases, Sisaket can serve as a model for identifying and understanding successful strategies and best practices to inform the scale-up of effective, appropriate, and acceptable approaches for malaria elimination, particularly in remote and border settings.

Understanding the unique RTA malaria risk profile, as well as any potential barriers hampering successful operationalization of the military’s response activities, is required to inform the necessary adaptation or tailoring of military-appropriate malaria diagnosis, treatment, and prevention activities. Towards this aim, a rapid formative assessment was undertaken in Sisaket Province in December 2021-January 2022, with the following objectives:

1. To understand and document RTA practices and perceptions related to malaria prevention, diagnosis and treatment, patient follow-up, and case-based surveillance;
2. To understand the perceived malaria risk and drivers of the recent malaria burden reduction in Sisaket Province;
3. To identify preferred product attributes for vector control tools and understand RTA perceptions on the safety, feasibility, and acceptability of existing and potential future interventions;
4. To identify opportunities and potential barriers for further RTA-MoPH coordination and collaboration on malaria elimination activities in Sisaket Province and Thailand.

## Methods

### Study setting and timeline

**Fig. 1:**
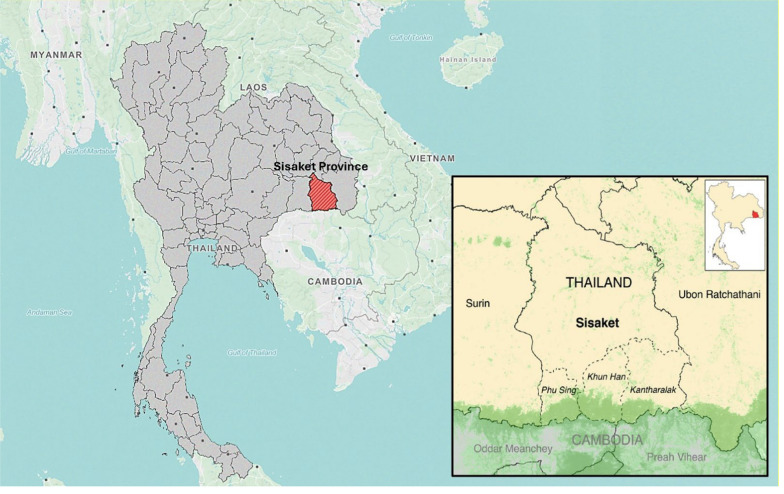
Map of Sisaket Province, Thailand

The formative assessment was conducted over a period of four weeks in December 2021-January 2022 in Sisaket Province’s three highest-burden districts, all situated along the heavily forested and disputed Cambodia border: Kantharalak, Khun Han, and Phu Sing (Fig. 1).

### Study context: malaria service delivery in military versus civilian health systems in Thailand

For civilian populations, the MoPH oversees the delivery of malaria services through two channels: (1) malaria-specific providers and clinics in the vertical malaria program under the Department of Disease Control’s (DDC) Division of Vector Borne Diseases (DVBD), and (2) the general health services structure (GHSS) through provincial, district, and subdistrict hospitals and malaria posts. In Sisaket, military health services are limited to a small field hospital in Kantharalak, while MoPH facilities include one DVBD malaria clinic located in Khun Han and one subdistrict health promotion hospital (HPH) and one district hospital in each of the three districts. All malaria diagnosis and treatment procedures at both MoPH and military facilities are standardized in accordance with the MoPH-issued national guidelines. Malaria is a notifiable disease in Thailand and every case must be reported by law to MoPH authorities. All cases diagnosed in both public and military sectors are reported into the DVBD’s Malaria Information System (MIS), a web-based platform that captures patient-level data.

### Study design, methods, and populations

Using a mixed-methods approach, the assessment targeted two study populations: (1) RTA personnel at subdistrict, district, provincial, and national levels (all ranks), and (2) MoPH staff at health facility, subdistrict, district, provincial, and national levels. The following data collection methods were employed:

1. Review and analysis of Sisaket’s retrospective MIS malaria case and program response data for 2016–2020 to identify recent trends in incidence, seasonality, and case classification, and characterize the RTA malaria risk profile
2. Focus group discussions (FGDs) conducted among:
  - RTA personnel organized into the following groupings:
    i. Private soldiers (voluntary or conscripted), who make up the largest RTA subgroup and are more likely to patrol than higher-ranking personnel
    ii. Non-commissioned officers (NCOs) and sergeants
    iii. Military medics
    iv. Military hospital staff (Kantharalak District only)
  - Local MoPH health facility-based staff (including hospitals and clinics)
3. Key informant interviews (KIIs) conducted among:
  - Senior RTA officials involved in malaria policy, administration, management, or service delivery at district, provincial, and national levels
  - Senior MoPH officials at district, provincial, and national levels.

A total of 15 FGDs and 24 KIIs among RTA and MoPH personnel were estimated to be required to achieve theoretical saturation (when interviews fail to produce new information). Pile sorting exercises with available vector control products were conducted as a group activity during FGDs to assess user preferences and identify opportunities for product optimization. Rooted in anthropology, pile sorting is a qualitative method used to explore and contextualize relationships among items within a specific domain (in this case, vector control products) to better understand stakeholder perceptions including the relative similarities, differences, and preferences among items. Commercially and locally available mosquito bite prevention products—including mosquito repellent lotions and sprays, mosquito coils, insecticide-treated nets (ITNs), and hammock nets—were used to facilitate discussions with RTA soldiers on the perceived product safety, effectiveness, ease of use, and suitability for forest use. Results from the pile sorting exercises including product rankings and notes on participants’ reasons for their selections and preferences were documented using a standardized pile sorting template. Data generated from these activities were incorporated into the FGD transcripts and qualitative datasets and analyzed alongside related FGD and KII data on malaria prevention practices and vector control product preferences.

### Study recruitment and eligibility

All FGD and KII participants were purposively recruited and required to meet the following eligibility criteria: (1) at least 20 years of age at the time of informed consent, (2) able and willing to provide written informed consent, (3) currently employed by the RTA, AMED, or MoPH, or stationed with the RTA in Sisaket Province, and (4) speaks the Thai language. RTA FGD participants in each district were organized according to their rank to help ensure the comfort of all participants in voicing their opinions. Higher-ranking RTA officials were interviewed one-on-one in KIIs and were not present during the RTA FGDs.

All participation in the FGDs and KIIs was entirely voluntary and without influence of military rank or chain of command. Informed consent was obtained from all FGD and KII participants in the local language, and each participant was provided with a signed copy of the consent form with study contact information. The informed consent form and semi-structured FGD and KII guides were developed in English, translated into Thai, and then back-translated into English to verify the accuracy of translations. Prior to implementation, the FGD and KII guides were field-tested with a small subset of RTA and MoPH staff and revised based on the findings. All members of the field team were trained on the formative assessment objectives, informed consent procedures, research ethics, qualitative interview methods, and the FGD and KII interview guides.

### Data analysis

The FGD and KII qualitative data transcripts were systematically reviewed and organized in Microsoft Excel spreadsheets and then coded and analyzed using an inductive content analysis approach, whereby emerging concepts and themes are identified, iteratively refined, and then grouped into broader categories and sub-categories to interpret the data and generate preliminary findings based on the assessment objectives.

Sisaket retrospective malaria case data for 2016–2020 were extracted from the MIS with all patient data deidentified to maintain confidentiality. Of note, the MIS combines military personnel and border police into a single occupational category; however, upon review of the 2016–2020 data, military bases were listed as the address for the vast majority of cases, and therefore all cases in this category are hereafter referred to as military cases (recognizing, however, that this is a potential source of misclassification bias, even if minimal). Data were organized in Microsoft Excel 2023 and descriptively analyzed using statistical methods to characterize the risk profile of military malaria cases (including age, gender, seasonality, case classification) and compare it to that of the non-military population. Available program response data on patient follow-up visits and implementation of the 1-3-7 surveillance strategy were also analyzed to identify and compare any trends among the military and non-military populations over this five-year period. To assess relationships between categorical variables, a Chi-square test of independence was performed, while a two-sample t-test assuming unequal variances was used to determine associations among continuous variables. Assumptions for all statistical tests were assessed and met, with the level of statistical significance set at a p-value of < 0.05 with 95% confidence intervals (CI).

## Results

A total of 16 FGDs (10 RTA and 6 MoPH) and 17 KIIs (8 RTA and 9 MoPH) were conducted, corresponding to a total of 116 respondents (72 RTA and 44 MoPH), as detailed in Table 1. The average duration of RTA and MoPH FGDs was 85 and 150 min, respectively, while that for RTA and MoPH KIIs was 53 and 80 min, respectively. Fewer KIIs than planned were conducted with RTA and MoPH national and district staff because theoretical saturation was felt to have been achieved and due to limited availability of some respondents owing to the demands of the ongoing COVID-19 pandemic.

**Table 1:** Number of RTA and MoPH FGDs and KIIs

District	RTA		MoPH	
	FGDs (# of participants)	KIIs	FGDs (# of participants)	KIIs
Kantharalak District	4 total (n = 28) • 1 Private soldiers (n = 7) • 1 Sergeants & NCOs (n = 6) • 2 Military hospital staff & military medics (n = 7; 8)	2	2 total (n = 13) • 1 HPH & DVBD clinic staff (n = 6) • 1 District hospital staff (n = 7)	2
Khun Han District	3 total (n = 18) • 1 Private soldiers (n = 8) • 1 Sergeants & NCOs (n = 6) • 1 Military medics (n = 4)	–	2 total (n = 11) • 1 HPH & district hospital staff (n = 7) • 1 DVBD clinic staff (n = 4)	2
Phu Sing District	3 total (n = 18) • 2 Private soldiers (n = 7; 7) • 1 Military medics (n = 4)	1	2 total (n = 11) • 1 HPH & DVBD clinic staff (n = 5) • 1 District hospital staff (n = 6)	2
Provincial level	–	3	–	3
National level	–	2	–	–
Totals	10 FGDs (n = 64)	8 KIIs (n = 8)	6 FGDs (n = 35)	9 KIIs (n = 9)

The results are organized into four sections:

1. Sisaket RTA malaria risk profile based on review of the five-year retrospective MIS case data;
2. Perceived malaria risk and drivers of the recent case reduction in Sisaket;
3. RTA practices and perceptions related to malaria prevention, diagnosis and treatment, patient follow-up, and case-based surveillance;
4. Opportunities and potential barriers to increased RTA-MoPH coordination and collaboration on malaria elimination activities.

### Sisaket RTA malaria risk profile

**Fig. 2:**
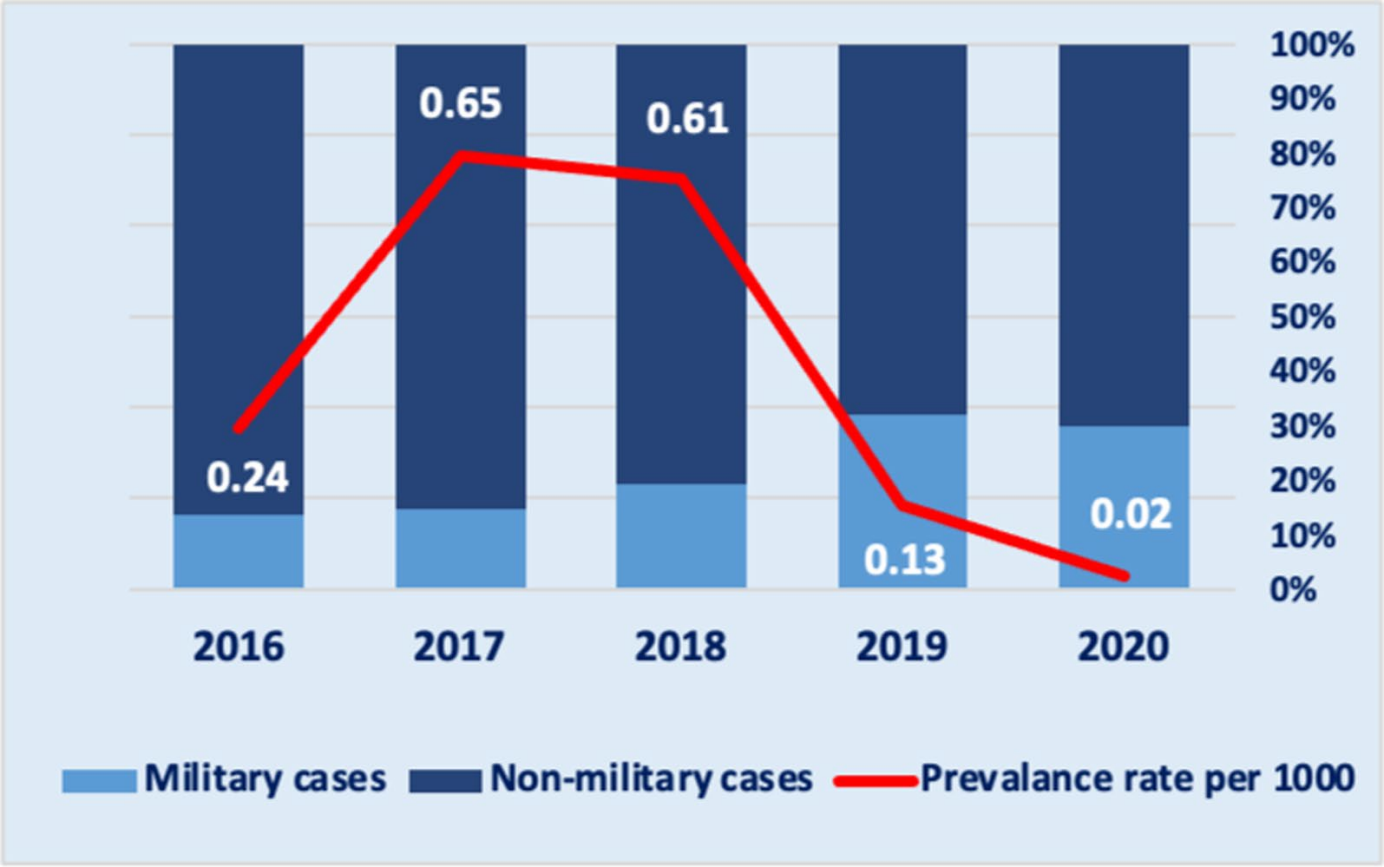
Sisaket Province malaria prevalence rate (per 1000) and proportion of military versus non-military cases 2016–2020

A total of 2425 malaria cases were reported in Sisaket Province between 2016 and 2020 with a mean prevalence rate (per 1000) of 0.33 (ranging from 0.02 in 2020 to 0.65 in 2017). The three study districts accounted for 94% of the total cases. Military patients made up 18% (432 cases) of total cases over the five-year period with the proportion increasing from 13% in 2016 to 32% in 2019 and 30% in 2020 (Fig. 2). As shown in Table 2, the proportions of *P. falciparum* (27% vs. 22%) and *P. vivax* (72% vs. 75%) infections were similar among military and non-military cases, with no statistically significant association observed.

**Table 2:** Characteristics of military versus non-military malaria cases 2016–2020 in Sisaket Province

Characteristics	Military malaria cases		Non-military malaria cases		Test statistics		
	n	Percent (%)	n	Percent (%)			
Total malaria cases	432	18	1993	82			
Gender							
Male	432	100	1747	88			
Female	0	0	246	12			
Age					t-value	df	p-value
Mean age	33		38		11.84*	664	< 0.001
Median	28		37				
Minimum	19		1				
Maximum	59		77				
Range	40		76				
Malaria species					Chi-square	df	p-value
* P. falciparum*	116	27	434	22	1.30**	1	0.254
* P. vivax*	306	72	1485	75			
Mixed	6	1	8	< 1			
* P. malariae*	0	0	3	< 1			
Unknown	0	0	63	3			
Seasonality							
Peak transmission season (May to September)	187	43	1110	56	3.93	1	0.047
Low transmission season (October to April)	245	57	882	44			
Case classification							
Indigenous	160	41	382	23	108.29	2	< 0.001
Imported within Thailand	73	19	1003	62			
Imported from abroad	158	40	248	15			

Bolded *p*-values denote statistically significant associations

*Two-sample t-test assuming unequal variances military vs. non-military ≥ 18 years old only

**Chi-square test comparing *P. falciparum* and mixed cases vs. *P. vivax, P. malariae,* and unknown cases

All 432 military cases (100%) diagnosed between 2016 and 2020 were male. The mean and median ages of a military case were 33 and 28 years respectively (range 19–59), with the mean age of non-military cases aged 18 years or older (40 years) significantly higher based on a two-sample t-test assuming unequal variances (*t-*value = 11.84, df = 664, *p*-value < 0.001). Based on a Chi-square test of independence, a significant but weak association was detected between seasonality of malaria infection and military vs. non-military status, with military patients more likely to be diagnosed during the low transmission season (October to April) than non-military patients (57% vs. 44%) (X^2^ = 3.93, df = 1, *p*-value = 0.047, N = 2424). While military personnel are stationed and patrolling in high-risk areas (such as the deep forest) in both low and high seasons, there may be less emphasis on malaria prevention during the low season and/or additional protection methods or better coverage during the rainy high season. A significantly higher proportion of military compared to non-military cases were classified as either imported from abroad (40% vs. 15%) or indigenous^1^ (41% vs. 23%) (X^2^ = 108.29, df = 2, *p*-value < 0.001, N = 2024).

### Perceived malaria risk and drivers of recent case reduction in Sisaket Province

The perceived level of local malaria risk in Sisaket Province varied by respondent type and district. Among RTA soldiers, Kantharalak participants reported a medium to high risk level (*‘It’s riskier than ever!’),* whereas those in Khun Han and Phu Sing districts felt it was low. Soldiers across all districts noted elevated risk during the rainy season and when operating in forest areas, highlighting the importance of self-protection measures to reduce risk. Among RTA and MoPH key informants, the majority reported low to moderate risk, though several perceived it to be high, citing poor awareness or adherence to protection measures among some soldiers (particularly new recruits), the recent establishment of new military bases requiring land clearing, and the increased risk of operating in forest and border areas.

Despite this, RTA and MoPH respondents across all three Sisaket districts felt that the local malaria transmission risk was declining. The perceived drivers of the declining malaria burden in Sisaket varied, with the most commonly cited including: increased implementation of proactive control measures (i.e., indoor residual spraying (IRS), community education, active case detection, patient follow-up, 1-3-7 strategy); reduced forest entry due to stricter military and forest ranger controls, COVID-19 restrictions and lockdowns, and fluctuating rosewood and rubber prices; greater adoption of self-protection measures, particularly ITNs and repellents, by both civilians and soldiers; and increased collaboration among local malaria partners on prevention efforts.

New soldiers, particularly privates and those originating from low-risk areas, as well as RTA forces recently deployed to new areas, were consistently identified as the highest-risk military subgroups. Most RTA soldiers reported that the malaria risk in Sisaket was higher than in their home province, attributing this to Sisaket’s forested landscape, extensive rubber plantations, and associated breeding sites. Some soldiers reported that prior to arriving in Sisaket and being trained on malaria, they had no prior knowledge of the disease and were initially more fearful of contracting it.

### RTA practices and perceptions related to malaria prevention, diagnosis and treatment, patient follow-up, and case-based surveillance

This section is structured into three subsection.

- Sect. 3.1 malaria prevention practices and perceptions, including preferred product attributes of vector control tools;
- Sect. 3.2 malaria diagnosis and treatment practices and perceptions; and
- Sect. 3.3 malaria patient follow-up and case-based surveillance practices and perceptions.

#### Malaria prevention practices and perceptions

RTA soldiers reported using similar personal protection measures at forward-operating bases and during forest patrols. RTA-issued mosquito repellent, available in both spray and lotion forms, was identified as the most widely used measure in both base and forest settings. At forward-operating bases, additional methods included ITNs, commercially available mosquito repellent sprays and lotions,^2^ mosquito coils, fans, blankets, insecticide spray, and long-sleeved clothing. While on forest patrols, soldiers also reported using other mosquito repellent sprays and lotions, hammocks and hammock nets, tents, and mosquito coils as protection. In addition to the RTA-issued repellent, soldiers in Phu Sing District reported using repellent supplied by the MoPH.

In pile sorting exercises, RTA soldiers and medics ranked repellents as the most effective tool for preventing malaria, with four of six FGDs identifying the RTA-issued repellent specifically, citing its long-lasting protection and effectiveness against other insects including red mites and ticks. Repellents were also rated as the easiest product to use in forest settings. Reported advantages included ease of application, portability, and long-lasting protection; while reported drawbacks included strong odor, stickiness (particularly when sweating), and skin irritation or allergic reactions (e.g., burning or itching sensations) in some users. Soldiers typically applied repellents in the evenings, before sleep, or when mosquitoes were perceived to be abundant.

Mosquito nets were ranked as the safest prevention product and the second most effective tool overall. Nets were favored by soldiers for their durability, ease of use, comfort, and availability at no cost. Reported concerns included fear regarding insecticide safety, uncertainty around retreatment of nets, discomfort using nets in warmer months, and the impracticality of using ITNs during forest patrols. Hammock nets were preferred for forest use given their portability, yet soldiers had to purchase these themselves, which was described as a financial burden. RTA-issued tents were available but considered too heavy and burdensome to carry on patrols.

Mosquito coils were used both indoors and outdoors, primarily in the evenings or during rest breaks when patrolling in non-border forest areas. While considered effective at repelling mosquitoes, coils were described as difficult to use in rainy or windy conditions, and had to be purchased at soldiers’ own expense. Many soldiers also noted that depending on their mission, mosquito coils were not always suitable for forest use as the smoke or smell could compromise their unit’s position.

Opinions were also sought on insecticide-treated clothing, a potential intervention under consideration by the RTA. Generally, the same key concerns were raised across all respondent types: risk of skin irritation owing to the insecticide chemical (noting that some soldiers had experienced allergic reactions to ITN insecticides); uncertainty around the clothing’s durability and insecticide potency after repeated washing; frequency of retreatment required; and likelihood of strong odors, especially during physical exertion. Despite identified safety and practical concerns, five of six soldier FGDs expressed openness to trying treated clothing if provided free of charge and shown through prior research to be safe. RTA respondents at district, provincial, and national levels expressed similar willingness to trial the intervention. Some participants highlighted that treated clothing could address an existing gap in malaria protection among soldiers, and several suggested that additional items beyond soldier uniforms be considered for treatment as well.

##### Prevention services offered by RTA medics and military hospital staff:

Prevention efforts by RTA medics focused on sanitation and environmental clean-up (e.g., eliminating breeding grounds, removing vegetation) as well as malaria education and reinforcement of adherence to personal protection measures among soldiers. Some medics also distributed RTA repellents, ITNs, or insecticide for net retreatment to soldiers. Although some key informants recalled medics also conducting IRS, this was not mentioned in the medic FGDs (perhaps because the medics interviewed were not aware or involved in this past activity), though medics expressed willingness to undertake IRS if supplied with the necessary equipment. Military hospital staff described providing more extensive prevention services, including IRS, trainings on net retreatment and repellent application, and distribution of insecticide to base medics for net retreatment.

#### Malaria diagnosis and treatment practices and perceptions

Between 2016 and 2020, 96% of military malaria cases (415 of 432) were diagnosed and treated at MoPH facilities: 74% at district or provincial hospitals; 18% at malaria clinics; 2% at subdistrict HPHs; and 2% at malaria posts (Table 3). The remaining 17 cases were diagnosed and treated at a military hospital.

**Table 3:** Treatment locations for military and non-military malaria cases in Sisaket Province, 2016–2020

Treatment location	Military population		Non-military population	
	n	Percent (%)	n	Percent (%)
MoPH district or provincial hospital	338	74	950	48
RTA military hospital	17	4	0	0
MoPH subdistrict HPH	9	2	142	7
MoPH malaria clinic	77	18	681	34
MoPH malaria post	8	2	220	11

RTA soldiers reported similar malaria care-seeking behaviors and practices across the three districts. At forward-operating bases, soldiers who develop a fever or suspect malaria first notify the medic and/or the base commander and are then referred to the nearest MoPH or military facility for malaria testing and treatment as these services are not available on site. Some soldiers indicated that when malaria symptoms are first suspected, medics provide antipyretics or other medications, and if symptoms persist after several days, the soldier is then referred off-base for testing by rapid diagnostic test (RDT) or blood smear microscopy, in accordance with Thailand’s national treatment guidelines.

Similarly, if a soldier develops a fever or suspects malaria while on patrol in the forest, they notify the medic and/or supervising officer who observes the symptoms and provides any necessary first aid; if symptoms do not improve, the soldier returns to the forward-operating base and is referred off-site for testing and treatment. If symptoms are severe and communication can be established, the soldier may be evacuated from the patrol area, but if contact cannot be made, the commander may have to abort the mission and withdraw all troops in order to safely escort the soldier back to base. Several soldiers across districts reported drinking an herbal remedy made from the root of the *Eurycoma longifolia* (or Tongkat Ali) tree at the first sign of fever or suspected malaria when in the forest (prior to any formal diagnosis). Participants in all soldier FGDs requested that testing and treatment services be available on site at military bases, citing the inconvenience of travel distance, poor road conditions, and time required to reach MoPH facilities.

##### Diagnosis and treatment services offered by RTA medics and military hospital staff:

The Kantharalak Military Hospital provides malaria testing and treatment to soldiers and requires that confirmed cases remain hospitalized for three days for observation and treatment monitoring. If a patient’s condition does not improve, the military hospital refers the soldier to the Kantharalak District Hospital. At the time of research, the military hospital doctor was deployed to the COVID-19 response and all soldiers were referred directly to the Kantharalak District Hospital, bypassing the military facility.

RTA medics confirmed that they do not provide any type of malaria testing or treatment on site at bases or during forest patrols. Several respondents indicated that RDT kits had previously been supplied to medics by AMED but expired before use and were not replaced. Medics in all districts expressed strong interest in receiving training on malaria testing, treatment, and active case detection, citing the benefits of prompt diagnosis and treatment as well as the logistical and security challenges associated with allowing non-military personnel to access bases to conduct these activities.

We don’t have any testing kits yet. If there are the test kits, it will be better than requesting support from external agencies because the problem will be solved immediately and without delay and the loss of personnel.

– RTA FGD participant

#### Malaria patient follow-up and case-based surveillance practices and perceptions

MoPH personnel reported conducting follow-up with RTA soldiers in accordance with national guidelines: follow-up visits with *P. falciparum* cases on days 3, 7, 28, and 42, and with *P. vivax* cases on days 14, 28, 60, and 90. The RTA including hospital staff and medics are not formally involved in follow-up activities with soldiers though some informal coordination takes place. The RTA defers to the MoPH patient follow-up and case-based surveillance strategies and protocols (no military-specific guidelines exist).

Between 2016 and 2020, the completion rate for all scheduled follow-up visits for both *P. falciparum* and *P. vivax* was 0% in both military and non-military populations. Completion rates for ≥ 1, ≥ 2, and ≥ 3 follow-up visits (any visit) across all malaria species were low but higher among military versus non-military cases: 39% versus 33% for ≥ 1 visit; 27% versus 14% for ≥ 2 visits (statistically significant); and 11% versus 10% for ≥ 3 visits. In both populations, follow-up visit completion rates increased annually from 2016 to 2019, with notable upticks in 2018 likely reflecting intensified response efforts following the 2017 outbreak.

Incomplete patient follow-up is not unique to military populations, though MoPH respondents across districts identified several military-specific challenges in conducting follow-up among RTA patients, namely: restricted access to military sites; difficulties contacting and tracking soldiers due to their mobility, rotation frequency, and deployment to areas with poor phone reception; and concerns about the privacy or safeguarding of location and personal information. Some MoPH staff proposed that military medics coordinate and conduct follow-up with soldiers, while RTA respondents recommended closer collaboration with the MoPH in this area, noting that base commanders could play a supervisory role in these activities.

As for the follow-up treatment, the DVBD cannot follow up with the soldiers. Therefore, the DVBD recommends that soldiers come for check-ups and that military medics coordinate the follow-up.

– MoPH FGD participant

For case-based surveillance, MoPH respondents reported implementing 1-3-7 activities in accordance with the national guidelines, using the LINE messaging application to notify district and provincial officials of new cases; the RTA is a member of the provincial LINE group. A reported operational workaround by the MoPH is to conduct the day 3 case investigation at the treating health facility on the same day as the day 1 diagnosis, provided the facility promptly reports the case and coordinates with the relevant MoPH staff. This workaround bypasses the need for MoPH staff to contact and follow up with the patient separately to conduct the case investigation interview within the three allotted days, thereby simplifying the operational logistics and improving the timeliness and completeness of the day 3 data.

Based on the 2016–2020 MIS data, the proportion of total military cases reported into the MIS within 24 h was 34%, compared to 43% among non-military cases (Table 4). Day 1 reporting rates in both populations showed an overall increasing trend over the five-year period, reaching 56% and 91% in 2020 for military and non-military populations, respectively. The proportion of total case investigations completed within three days was 89% among military patients versus 81% among non-military patients—a slight but statistically significant difference (X^2^ = 4.10, df = 1, *p*-value = 0.043, N = 2386). Case investigation rates in both populations increased annually from 2016 to 2020, with both groups achieving 100% completion within three days in 2020.

**Table 4:** Day 1 reporting and day 3 case investigation rates for military and non-military populations in Sisaket Province, 2016–2020

	Day 1 reporting rate				Day 3 case investigation rate			
Year	Military		Non-military		Military		Non-military	
	n	Percent (%)	n	Percent (%)	n	Percent (%)	n	Percent (%)
2016	13	27	102	33	32	67	178	58
2017	17	12	239	29	124	88	661	81
2018	75	43	411	57	164	94	634	87
2019	37	63	87	69	56	95	120	95
2020	5	56	19	90	9	100	21	100

Day 7 response data could not be disaggregated by military versus non-military populations, as these activities are implemented at village level and the corresponding data recorded in aggregate, precluding comparison of day 7 completion rates by populations. For indigenous RTA cases, MoPH staff require special permission to enter military bases to conduct day 7 activities, though respondents noted this process had improved over time as MoPH staff developed better working relationships with RTA authorities. No response measures can be undertaken for imported cases contracting malaria abroad.

### Opportunities and potential barriers to increased RTA-MoPH coordination and collaboration on malaria elimination activities

All key informants were asked about existing RTA-MoPH coordination efforts and associated challenges across specific program areas, with the aim of identifying potential opportunities and anticipated barriers to further collaboration and support.

#### General RTA-MoPH coordination and collaboration on malaria activities

RTA respondents reported some level of coordination with the MoPH at national, provincial, district, and local levels. While the scale and scope of RTA-MoPH coordination varied by district, all districts reported that coordination occurred through both formal and informal channels, with some respondents noting that collaboration had expanded or improved in recent years.

Nowadays, it is easier to connect and coordinate. The military began to open up some areas for the MoPH to take part in some activities, and the MoPH gave great support.

– RTA key informant

Coordination took the form of in-person meetings (primarily at provincial level), telephone and LINE communications, and exchange of official documents via the postal system. Several RTA respondents referenced a 2020 memorandum of understanding (MOU) with the MoPH on malaria reduction and elimination, underscoring the importance of clearly defined partner roles, responsibilities, and guidelines for collaboration. Depending on the nature of the activity, MoPH staff reported coordinating directly with RTA senior-ranking officials as well as military medics at the operational level.

The key coordination challenges identified by RTA and MoPH respondents included: frequent RTA staff rotations disrupting continuity of relationships; communication barriers including lack of access to contact information and poor phone signal in some areas; restricted MoPH access to military sites; gaps in coordination among multiple partners operating in high-risk border areas; limited malaria knowledge or prioritization among some RTA and MoPH staff from non-endemic areas; and budgetary constraints or inefficiencies across ministries.

Despite these challenges, both RTA and MoPH respondents expressed strong interest and willingness to strengthen military-civilian coordination, proposing several recommendations to support these efforts: designating dedicated RTA and MoPH focal points or liaisons at each level; ensuring the MoPH is informed of RTA personnel rotations with updated contact information; establishing an MOU to formalize joint operating procedures and clearly delineate roles and responsibilities (for example, the MOU could stipulate broader terms for MoPH access to military sites, eliminating the need for individual permission letters for each activity); and increasing communication through routine RTA-MoPH touchpoints to promote more advanced planning, improved information sharing, and earlier identification of support needs.

#### Malaria policy, guidelines, and financing

The RTA defers to relevant MoPH-issued malaria policies, guidelines, and standard operating procedures (SOPs), as apart from the AMED prevention policy focused on preventive medicine and sanitary care, no military-specific malaria strategies or guidelines exist. Several RTA respondents mentioned participating in provincial-level MoPH annual or semi-annual policy and planning meetings, but respondents across districts identified the need for additional RTA-MoPH collaboration in malaria policymaking and guideline development. RTA respondents expressed interest in further engaging with the MoPH at national level on malaria strategy and policy development and deemed valuable any potential support in adapting or establishing military-specific guidelines if required.

With respect to financing, the RTA does not maintain a dedicated malaria budget, rather malaria expenses are covered under the military’s general preventative medicine budget, with individual RTA units responsible for managing their own malaria-related expenditures. MoPH respondents were often unaware of this arrangement, assuming the military had its own malaria-specific funding. Both RTA and MoPH cited budget constraints as a challenge and identified joint planning and resource pooling as important strategies for improving cost efficiency and program integration.

#### Malaria prevention

All three districts identified the MoPH (DVBD) as playing some role in the provision of protective equipment to the RTA including ITNs, insecticide for retreatment, and IRS, but the type and regularity of support varied by and within districts. Otherwise AMED was reported to supply RTA units with the required protective equipment including ITNs and repellents. Yet certain supplies—namely IRS equipment—cannot be procured using military funds, rendering MoPH support in this area essential. Several MoPH respondents noted that without explicit supply requests from the RTA, it is assumed that the military has sufficient stocks of equipment.

The MoPH (DVBD) leads coordination and response efforts during malaria outbreaks, including IRS spraying and providing any additional equipment required by the RTA. In one district, routine IRS spraying at military sites occurred approximately twice a year upon RTA request, while the other two districts reported that IRS was only implemented during outbreaks despite requests for more routine spraying.

During the time the spray was needed, the Colonel would make a request to the HPH for support. But during this period there was no outbreak, so there was no spraying.

– RTA FGD participant

Training on malaria prevention for military personnel in Sisaket is provided by both the RTA and MoPH. In line with AMED policy, all ranks including new recruits receive an annual training focused on disease prevention and self-protection (including against malaria) approximately one month prior to deployment. These trainings are facilitated by both military hospital staff and MoPH personnel with support from the Raks Thai Foundation. Some RTA respondents also referenced mid- and post-deployment trainings, while others indicated that these were planned for future implementation. MoPH staff across districts reported providing additional ad-hoc training in response to specific RTA requests—including demonstrating use of protection methods and supplying training and media materials.

A consistent challenge reported by both RTA and MoPH respondents was insufficient vector control equipment, specifically shortages of ITNs, permethrin for net retreatment, and repellents. Frequent rotations of soldiers, who reportedly take the equipment with them when transferred to new bases, contributed to this issue; in response, it was suggested that all equipment be stored centrally at each base. RTA respondents identified the following priority areas for additional MoPH support in malaria prevention: regular supply of ITNs and IRS equipment, routine (non-outbreak) IRS at military sites, and clearer guidelines for prevention and supervision practices.

#### Malaria testing, treatment, and surveillance

In all three districts, the MoPH coordinates with the RTA to conduct active case detection at military sites during malaria outbreaks and as part of the day 7 response measures; only one district reported ongoing active case detection in non-outbreak settings. RTA respondents in all districts requested that active case detection be conducted more routinely on military bases. Both RTA and MoPH respondents recommended that medics be trained and equipped to conduct on-site malaria testing and active case detection at military sites. MoPH officials offered to provide the required training, emphasizing the need for prompt diagnosis and treatment especially along remote border postings. The MoPH trains RTA hospital staff on blood draws and malaria slide examination and provides all malaria testing and treatment equipment to the Kantharalak Military Hospital.

If the military can recruit military medics to take care of the health of small bases along the border, the MoPH can go in for training on blood testing and rapid testing immediately. So, they don't have to wait – if they suspect an infection, they can test it.

– MoPH key informant

## Discussion

Characteristic of elimination settings, military cases comprised an increasing proportion of Sisaket Province’s declining malaria burden between 2016 and 2020; a reduction perceived to be largely attributable to: implementation and scale-up of more proactive prevention, surveillance, and response measures; reduced forest exposure due to stricter controls on forest entry; increased adoption and adherence to personal protection methods; and greater coordination and collaboration among local malaria stakeholders. All 432 military cases diagnosed in Sisaket during this five-year period were male, and on average these patients were significantly younger, more likely to be classified as either imported or indigenous cases, and more likely to be infected during the low transmission season as compared to their non-military counterparts.

RTA soldiers across the three districts reported using similar malaria prevention methods, with the RTA-issued repellent and ITNs identified as the most widely used and preferred tools; of note, these were the two tools provided at no cost and therefore soldiers likely had the most experience and familiarity with them. Soldiers demonstrated a strong understanding of malaria including risk factors and the importance of self-protection, suggesting that RTA training efforts are robust and effective. However, with 57% of military cases diagnosed during the low season, the recommendation for year-round protection may need to be reinforced. Preferred product attributes for vector control tools included: ease of use, portability, durability, non-irritating, effectiveness against other insects, and cost-free. Findings from this assessment underscore the critical need for more appropriate and targeted vector control interventions for forest-going and military populations. Given the unique operational security requirements of military operations, product design specifications should include: no detectable odor, absence of smoke or visible vapor, silent operation, low-visibility or camouflaged coloring, and durability under rugged conditions.

Malaria care-seeking practices among the RTA in the three districts were largely aligned, with 96% of military patients diagnosed and treated at MoPH facilities. Malaria testing and treatment services are not available on site at military bases or during forest patrols; though medics expressed strong interest in being trained on these activities, which could prove particularly valuable in high-risk border postings, and given the high rate of imported cases. With the RTA primarily focused on malaria prevention, MoPH personnel conduct all patient follow-up and 1-3-7 case-based surveillance and response activities in Sisaket, though actual implementation may be suboptimal given the often-remote military postings. Follow-up visit completion rates remained low among both military and non-military populations, though these efforts may become more manageable as malaria caseloads decline. While day 1 reporting rates increased annually over the five-year period, reporting of military cases was still comparatively lower than that of non-military cases—56% vs. 91% in 2020 respectively; although reasons for this discrepancy were not explored, late reporting among military populations can delay treatment and response measures, resulting in onward malaria transmission among troops. The MoPH achieved high rates of case investigations in military patients, with 89% of cases investigated within three days between 2016 and 2020, which was partly credited to the practice of conducting case investigations on day 1 at the health facility.

RTA-MoPH coordination on malaria response efforts was reported to occur at all levels using both formal and informal channels. Some respondents expressed that collaboration was expanding and improving, although certain areas could benefit from increased coordination, specifically: malaria policymaking and guideline development; vector control; patient follow-up and active surveillance; and control efforts along high-risk border areas. The RTA and MoPH both expressed strong interest and willingness to strengthen their partnership and engagement, but as caseloads decline coordination challenges become increasingly consequential and must be addressed, namely: lack of relationship continuity given the frequency of RTA rotations; restricted MoPH access to military sites; communication barriers; and broader differences in military versus civilian operating procedures.

This formative assessment was subject to several limitations. First, the research was conducted during the COVID-19 pandemic, which impacted the local study team’s and key informants’ availability (including no KII participation by the national-level MoPH) and precluded any in-person meetings with the full study team. Second, if recently transferred from another military post, the interviewed RTA respondents may have been unaware of historical RTA-MoPH collaboration on malaria; moreover, joint efforts were likely at a low during the COVID-19 pandemic when data collection occurred. Third, despite steps to mitigate social desirability bias especially among military hierarchies, some participants may have tailored their FGD and KII responses to appear more favorable, appease higher-ranking officials, or align with accepted or established norms, which can threaten internal data validity and distort key qualitative findings. Fourth, and inherent to qualitative research, the generalizability of these findings is likely limited, even within Thailand, due to the reported variability in some malaria practices across the three districts and the pre-existing RTA-MoPH partnership in Sisaket owing to the 2017 outbreak and response. Furthermore, while this research focused on primarily qualitative data, incorporating supplementary quantitative data could further enrich future studies. Lastly, while theoretical saturation was achieved, expanding KIIs to include national-level MoPH officials in future assessments could provide additional strategic insights.

## Recommendations

To promote further RTA-MoPH coordination towards controlling and eliminating malaria in Thailand, several key recommendations emerged:

### Establish a broad-reaching MOU between the RTA and MoPH with strong political buy-in

The MOU could clearly define partner roles and responsibilities, areas of coordination and support (based on risk stratification level), routine and ad-hoc coordination and communication procedures (e.g., meeting frequency), data-sharing mechanisms, confidentiality clauses, an overarching permission to better facilitate MoPH access to military sites (e.g., specifying certain timeframes for entry based on support plans), and any other terms of engagement. Such an MOU would require advanced planning of joint activities, which the RTA requested, as well as promote greater standardization, transparency, and budget efficiencies. A specific RTA malaria budget could also be developed based on the advanced planning and agreed scope of collaboration.

### Identify designated RTA and MoPH focal points at national, provincial and district levels

As expressed by respondents, having specific RTA and MoPH focal points at the different levels, and ensuring handover of new personnel information, would foster better coordination and communication, given the frequent turnover due to military rotations. The focal points could help ensure adherence to the terms defined in the MOU and increase RTA-MoPH touchpoints through routine meetings or other specified communication channels.

### Capacitate RTA medics to conduct standardized prevention, diagnosis, treatment, patient follow-up, and other surveillance activities, adapting the methods as required by the military

MoPH and RTA respondents alike recommended further training military medics to enable them to carry out prevention (including IRS), diagnosis and treatment, patient follow-up, surveillance (including active case detection), and training activities for RTA soldiers. Transferring these skills to medics, while ensuring uninterrupted supplies of the required equipment, would allow for earlier diagnosis and treatment and improved patient follow-up and treatment adherence among soldiers, as well as address the MoPH staffing constraints and restricted access to military sites. Medics could serve as an on-site malaria resource for soldiers, increasing their confidence and sense of empowerment in protecting themselves against malaria. Some MoPH protocols may need to be adapted to accommodate the unique military circumstances, and supervision and oversight by more senior-ranking officers may also be required.

## Conclusion

Findings from this study extend the very limited knowledge base on military malaria practices and military-civilian collaboration in malaria control and elimination efforts. More operational research and documentation is needed to understand how militaries and national malaria programs can best work together to protect their forces from malaria and advance more rapidly towards the shared goal of elimination. Given military intelligence, operational security, and often remote locations, as well as limited participation in peer-reviewed literature, joint military-civilian research efforts may require some creativity or negotiation, but this study serves as a successful example. As demonstrated by the recent resurgence along the Thai–Myanmar border, gains in malaria control remain fragile, underscoring the need for robust, vigilant surveillance and resilient, responsive health systems—both of which require clear, strong, and efficient coordination among all malaria stakeholders. With military personnel frequently deployed along high-risk international borders, they remain particularly vulnerable and are often among the first affected by rising transmission levels; but they are also strategically positioned to support surveillance and response efforts in these often hard-to-reach areas. As high-risk populations are increasingly targeted in the Greater Mekong Subregion, it is essential that militaries be effectively engaged as both a risk group and elimination partner if national and regional malaria elimination is to be achieved and successfully sustained.

## Data Availability

The data generated and analyzed in the current study may be available from the corresponding author upon reasonable request and with permissions from the Thailand MoPH and/or the RTA.

## Abbreviations

AFRIMS: Armed Forces Research Institute of Medical Sciences
AMED: Army Medical Department, Royal Thai Army
CI: Confidence interval
DDC: Thailand’s Department of Disease Control
DVBD: Thailand’s Division of Vector Borne Diseases
FGD: Focus group discussion
GHSS: General Health Services Structure
HPH: Health Promotion Hospital
IRS: Indoor residual spray
ITN: Insecticide-treated net
KII: Key informant interview
MIS: Thailand’s Malaria Information System
MoPH: Ministry of Public Health
MOU: Memorandum of understanding
NCO: Non-commissioned officer
NMES: Thailand’s National Malaria Elimination Strategy 2017–2026
RDT: Rapid diagnostic test (malaria)
RTA: Royal Thai Army
SOP: Standard operating procedure
UCSF MEI: University of California, San Francisco’s Malaria Elimination Initiative

## Footnotes

1 An indigenous case is defined as a patient who contracted malaria in the village where the patient lived during the infection period [7].

2 Commercially available repellent brands reported by respondents included September 15, Soffel, and Sakura.
